# Effects of Alternative Prey Density on Cannibalism in Adult Female *Neoseiulus californicus*

**DOI:** 10.1002/ece3.73519

**Published:** 2026-04-28

**Authors:** Fan‐Xue Zhang, Xiang‐Zhi Chen, Feng Xiao, Jia‐Yun Zhu, Rong Xiao

**Affiliations:** ^1^ Guizhou Key Laboratory of Agricultural Biosecurity, Institute of Entomology Guizhou University Guiyang People's Republic of China; ^2^ State Key Laboratory for Biology of Plant Diseases and Insect Pests Institute of Plant Protection, Chinese Academy of Agricultural Sciences Beijing People's Republic of China

**Keywords:** alternative prey density, cannibalism, *Neoseiulus californicus*, strain

## Abstract

*Neoseiulus californicus* is a key predatory mite species used for controlling pest mites on crops such as strawberries and corn. Cannibalism is common among predatory mites, but studies on how prey density influences the cannibalistic behavior of *N. californicus* remain limited. To clarify the regulatory effects of alternative prey density, rearing strain, and conspecific life stage, this study used *Oulenziella bakeri* (at four densities: 0, 1, 3, and 5 individuals) as the alternative prey, and compared the cannibalism of adult females of two strains of *N. californicus* , namely TU (reared long‐term on *Tetranychus urticae*) and OB (reared long‐term on *O. bakeri* ), toward conspecific eggs, larvae, protonymphs, and deutonymphs (cannibalism primarily occurs when mature female adults prey on conspecific immature stages). The results showed that *O. bakeri* density had no significant effect on the cannibalism rates of either strain (*p* > 0.05). However, the cannibalism rate of the TU‐strain toward larvae and protonymphs showed a downward trend, while the cannibalism rate of the OB‐strain on eggs decreased significantly with increasing *O. bakeri* density. Both strains exhibited a preference for larvae. Rearing strain and life stage were significant factors influencing the probability of cannibalism, and a significant interaction was observed between them. Specifically, the OB‐strain showed significantly lower cannibalism than the TU‐strain only toward eggs, although it also displayed a lower (non‐significant) cannibalism trend across other life stages. These findings provide a basis for optimizing population stability and production efficiency in mass‐rearing systems.

## Graphical abstract

Using two strains of *N. californicus* —TU (reared long‐term on *T. urticae* ) and OB (reared long‐term on *O. bakeri* )—this study demonstrated that although alternative prey density had no significant effect on cannibalism, both rearing strain and conspecific life stage significantly influenced cannibalistic behavior, with a significant interaction between these factors. Only toward eggs did the OB‐strain show significantly lower cannibalism than the TU‐strain, although it also displayed a lower (non‐significant) cannibalism trend across other life stages. These findings provide important insights for optimizing mass‐rearing systems by considering strain‐specific cannibalistic tendencies.

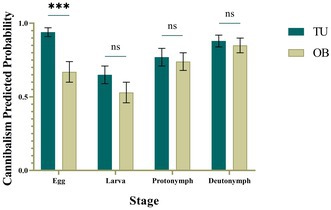

## 1 Introduction

Phytoseiid mites (Acari: Phytoseiidae) are important predatory mites in agricultural ecosystems. These mites effectively control small arthropod pests such as spider mites, thrips, whiteflies, and aphids, making them widely used as biological control agents globally (Helle and Sabelis 1985; Van Lenteren 2012; Yuan et al. 2024). Their control efficacy has been verified by numerous studies; for example, *Typhlodromalus aripo* successfully controls the cassava green mite *Mononychellus tanajoa* (Acari: Tetranychidae); *Euseius stipulatus* effectively fights harmful mites on citrus and avocado crops; and *Neoseiulus arundonaxi*, *N. barkeri* , *N. bicaudus*, and *Cydnoseius negevi* also exhibit excellent pest control effects against the western flower thrips *Frankliniella occidentalis* (Thysanoptera: Thripidae) (Onzo et al. 2003; Palevsky et al. 2013; Sanad and Hassan 2019).

Despite the outstanding pest control efficacy of phytoseiids, their field application is often constrained by their behavioral traits. Among these traits, cannibalism (i.e., intraspecific predation between individuals of the same species) is one of the key factors that reduce the effectiveness of their application as natural enemies (Polis and Holt 1992; Tang et al. 2004). Phytoseiids could exhibit cannibalistic or interspecific predatory behavior (the predation between different species) when prey availability is limited. Specifically, *N. californicus* has a higher interspecific predation rate than most Type III predatory mites, i.e., generalist predatory mites with a broad dietary range that can effectively prey on various small arthropods, particularly phytophagous spider mites (Walzer et al. 1999a, 1999b).

Food is a key factor influencing cannibalism in predatory mites (Marcossi et al. 2020). In natural ecosystems, *N. californicus* primarily preys on phytophagous spider mites, with *Tetranychus urticae* serving as its typical natural prey. The two species have developed a tightly linked predator**–**prey relationship through long‐term coevolution (Mu et al. 2025). In biological control practice, an alternative food source is often used to improve the efficiency of large‐scale natural enemy rearing or to adapt to specific crop environments. This type of prey is easy to mass‐rear and nutritionally suitable. For example, *Oulenziella bakeri* (Acari: Winterschmidtiidae), employed in this study, has become an important alternative prey due to its rapid reproduction, low rearing cost, and ability to support normal development of predatory mites (Rasmy et al. 1987; Mu et al. 2025). When natural prey is insufficient, predatory mites can maintain survival and reproduction by feeding on alternative prey (e.g., other mites, insect eggs) or alternative food sources (e.g., pollen), and high‐quality resources can inhibit cannibalism. Studies have shown that when adult *Amblyseius herbicolus* are fed *O. bakeri* and *T. urticae* , there is no cannibalistic behavior of consuming conspecific eggs (Hou et al. 2022). Marcossi et al. (2020) observed that in the presence of high‐quality food (cattail pollen), cannibalistic behavior of *A. herbicolus* toward conspecific eggs decreased significantly. Narrow‐leaved cattail pollen could significantly reduce cannibalistic behavior of *E. stipulatus*, as well as the predatory behavior of *Iphiseius degenerans* toward *E. stipulatus* (Calabuig et al. 2017). However, not all high‐quality foods can effectively alleviate this behavior. For example, in the presence of pine pollen, the cannibalistic behavior of *Amblyseius eharai* and *E. stipulatus* did not significantly decrease (Calabuig et al. 2017; Tsuchida et al. 2022).

Studies have shown that the survival rate of *N. californicus* reared on *O. bakeri* is higher compared to that on its natural prey, *T. urticae* (Zhu et al. 2023). However, higher survival rates may increase population density, potentially intensifying intraspecific competition. This highlights the need for further investigation into its cannibalistic behavior in *N. californicus* under different prey conditions. Existing studies have demonstrated that under high prey densities (e.g., *T. urticae* ) that exceed a specific threshold, the predation efficiency of *Amblyseius swirskii* decreases significantly (Elmoghazy et al. 2024). In contrast, under conditions of low natural prey density, the cannibalism of *Phytoseiulus persimilis* increases significantly (Xu et al. 2023). Farazmand et al. (2014) found that when the primary prey, *T. urticae* , was abundant, cannibalism rates in *N. californicus* were low or even absent; however, in the absence of the primary prey, cannibalistic behavior of *N. californicus* intensified. These findings confirm a clear association between prey density and the functional response of predatory mites. However, whether this relationship holds true for the effects of alternative prey density on cannibalism of *N. californicus* remains unclear.

Current research also suggests that predatory mites may exhibit cannibalistic preferences for conspecific individuals at different life stages. For example, when prey is scarce, cannibalism by *P. persimilis* toward nymphs increases significantly (Xu et al. 2023). However, it remains unclear whether *N. californicus* shows similar stage‐specific preferences—for example, toward eggs, larvae, protonymphs, or deutonymphs—or whether such preferences are influenced by prey density and rearing strain. Although progress has been made in applied research on *N. californicus* , significant knowledge gaps remain regarding the regulatory mechanisms underlying its cannibalistic behavior. Specifically, these include: the influence of alternative prey density on cannibalism rates in different strains; whether stable behavioral differences exist between strains long‐adapted to distinct prey sources; and how the mite's feeding preference for conspecific life stages is modulated by prey availability. To address these gaps, we used *O. bakeri* as alternative prey and established a density gradient to systematically compare cannibalism by two strains—TU (reared long‐term on *T. urticae* ) and OB (reared long‐term on *O. bakeri* )—toward conspecific eggs, larvae, protonymphs, and deutonymphs. This study aims to clarify the effects of alternative prey density, rearing strain, and their interaction on cannibalistic behavior, thereby providing a theoretical basis for optimizing mass‐rearing and field population management of *N. californicus* .

## 2 Materials and Methods

### 2.1 Experimental Mite Source

#### 2.1.1 Breeding of TU‐Strain

A laboratory‐reared population of *T. urticae* was originally collected from *Phaseolus vulgaris* (Fabales: Fabaceae) field in Huaxi District, Guiyang City, Guizhou Province, and has been maintained on kidney beans in the laboratory for more than 3 years. Kidney beans were grown in an artificial climate chamber to serve as hosts. When the seedlings had developed 3–4 true leaves, female and male adults of *T. urticae* were inoculated onto the seedlings using a fine brush for mass rearing. *Neoseiulus californicus* were obtained from Fuzhou Guannong Biotechnology Co. Ltd. A strain of *N. californicus* was reared on this *T. urticae* population in the laboratory for more than 1 year to establish a *T. urticae* ‐reared strain (designated as the TU‐strain) (Zhu et al. 2023).

#### 2.1.2 Breeding of OB‐Strain

*Neoseiulus californicus* and *O. bakeri* were obtained from Fuzhou Guannong Biotechnology Co. Ltd. A colony of *O. bakeri* was established and reared on yeast granules (Angel Yeast Co. Ltd.) as a food source, and has been maintained on kidney beans in the laboratory for more than 3 years. The purchased *N. californicus* colony was transferred to an artificial climate chamber and maintained on *O. bakeri* under laboratory conditions for more than 1 year to establish an *O. bakeri* ‐reared strain of *N. californicus* (designated as the OB‐strain) (Zhu et al. 2023). *Neoseiulus californicus*, *O. bakeri* , and *T. urticae* were all reared under the following environmental conditions: temperature, 25°C ± 1°C; relative humidity, 75% ± 5% RH, and photoperiod of 16 L:8 D.

### 2.2 Rearing Device and Experimental Device

#### 2.2.1 Rearing Device

**FIGURE 1:**
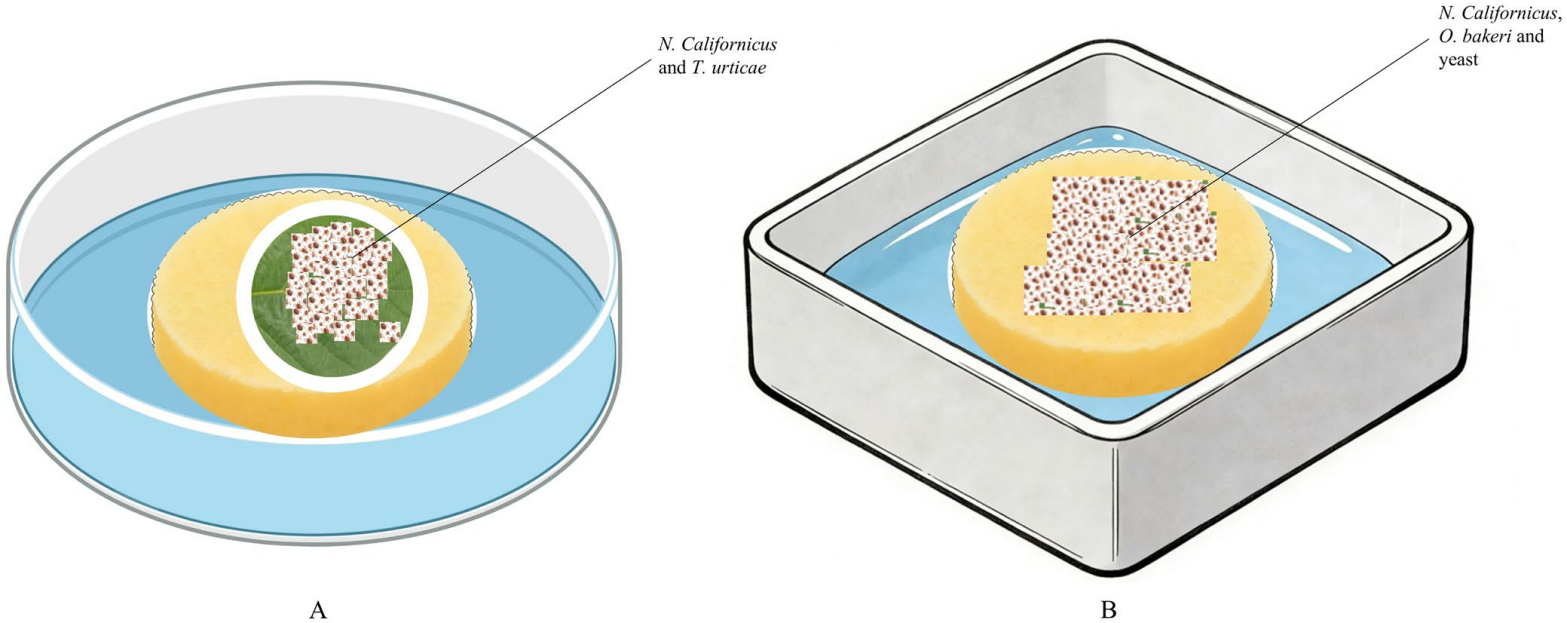
Schematic diagram of the rearing apparatus for the experimental mite colonies (The rearing setup for A: TU‐strain; B: OB‐strain).

The rearing setup for the TU‐strain used a Petri dish (15 cm diameter, 2 cm height) containing a hydrated sponge (14 cm diameter, 1.5 cm thick). A kidney bean leaf was placed with the adaxial surface down on the sponge. A water barrier was constructed by surrounding the leaf edge with moist absorbent cotton to confine the mites, while additional moist cotton was wrapped around the petiole to maintain leaf turgor. All developmental stages of *T. urticae* were transferred to the abaxial surface of the leaf. Subsequently, *N. californicus* was introduced onto the leaf, and additional *T. urticae* were added daily to sustain the predator population. Water was added to the Petri dish to maintain the water level flush with the sponge, thereby ensuring high humidity (Figure 1A). The kidney bean leaves were replaced daily to provide a fresh environment, and any withered leaves were removed promptly (Zhu et al. 2023).

The rearing setup for the OB‐strain consisted of an 18 × 18 × 8 cm plastic box. A sponge (diameter > 10 cm) was placed at the bottom of the box, surmounted by a 10‐cm‐diameter Petri dish containing an excess of yeast to provide ad libitum food for *O. bakeri* . The yeast was replenished every 3 days. To prevent mite escape, water was added to the bottom of the box to create a moat, with the water level maintained flush with the top of the sponge. Once a stable population of *O. bakeri* was established, *N. californicus* was introduced into the Petri dish (Figure 1B). Thereafter, additional *O. bakeri* were added daily to sustain the predator population (Zhu et al. 2023).

#### 2.2.2 Experimental Device

The test chamber was an enclosed space (40 × 30 × 8 mm) constructed from three transparent acrylic plates and one kidney bean leaf, assembled in the following order from top to bottom: (1) a plate (40 × 30 × 2 mm); (2) a plate (40 × 20 × 3 mm) featuring a central circular hole (20 mm in diameter); (3) a kidney bean leaf placed with its adaxial surface facing down; and (4) a plate (40 × 30 × 2 mm). The outermost (first and fourth) acrylic plates were clamped together with two binder clips, forming a relatively enclosed space in the middle of the device for mite experiments.

### 2.3 Experimental Procedures

Newly emerged adult females of each *N. californicus* strain (which are significantly longer in body length than males) at the same developmental stage were collected using a fine brush under a stereomicroscope and then starved for 24 h prior to use in the experiment. Since cannibalism in *N. californicus* mainly occurs when adult females prey on conspecific immature stages, the following experimental combinations were designed (Table 1).

**TABLE 1:** Experimental design combinations for cannibalism by adult females of *N. californicus* (TU and OB strains) on conspecific immature individuals.

Predator/individual		Prey/individual		Prey density/individual			
Strain	Stage	Strain	Stage	Density 1	Density 2	Density 3	Density 4
*Neoseiulus californicus* (TU/OB)		*Neoseiulus californicus* (TU/OB)		*Oulenziella bakeri*			
	Female adult		Egg	0	1	3	5
	Female adult		Larva	0	1	3	5
	Female adult		Protonymph	0	1	3	5
	Female adult		Deutonymph	0	1	3	5

The cannibalistic behavior of TU‐strain adult females was tested against four conspecific immature stages: eggs, larvae, protonymphs, and deutonymphs. For each stage, one starved female was placed in the test arena with one individual of the respective immature stage. To test the effect of alternative prey density, we released *O. bakeri* adults at one of four densities: 0, 1, 3, or 5 individuals per arena. These numbers corresponded to area densities of 0, 0.32, 0.96, and 1.59 individuals per cm^2^, respectively, given a circular arena with a diameter of 20 mm (area ≈3.14 cm^2^). Each of these treatment combinations was replicated 20 times. After 24 h, we recorded whether cannibalism had occurred (1 = yes; 0 = no). Any replicate in which mites escaped was discarded and excluded from the analysis.

The cannibalism experiment with female adults of the OB‐strain was conducted in the same manner as that with the TU‐strain.

### 2.4 Data Analysis

The raw data were processed and summarized using Excel 2019, with the cannibalism rate calculated as (number of cannibalism events/total replicates) × 100%. Statistical analysis was performed using SPSS 27.0. A binomial generalized linear model (GLM) was fitted, with the occurrence of cannibalism (yes/no) as the dependent variable and alternative prey density, rearing strain, and conspecific life stage as independent variables. The model included the main effects of all independent variables and the interaction term between rearing strain and conspecific life stage (strain × stage). The significance of each effect was assessed using the likelihood ratio test, and parameters were estimated via Fisher's scoring algorithm. For the significant interaction between rearing strain and life stage, pairwise comparisons were performed to examine differences between strains within each life stage. Based on the GLM results, the data were statistically summarized and visualized using GraphPad Prism 8.0.2.

## 3 Results

### 3.1 Cannibalism Rates of TU‐Strain and OB‐Strain Female Adults Under Different Densities of Alternative Prey

**FIGURE 2:**
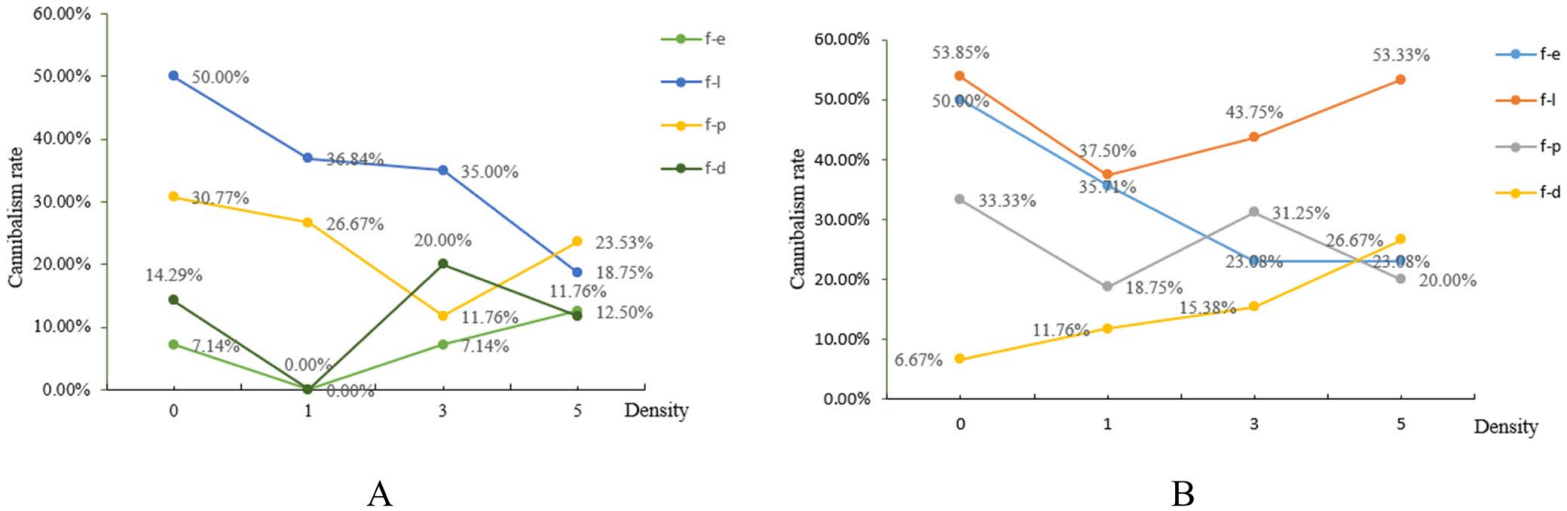
Effect of prey density on cannibalism rates of adult females of the TU and OB strains (A: TU‐strain; B: OB‐strain). d, deutonymph; e, egg; F, female adult; l, larva; p, protonymph.

When the prey density increased from 0 to 5 individuals per arena, the cannibalism rate of TU‐strain females on conspecific eggs increased from 7.14% to 12.50%. In contrast, their cannibalism rates on conspecific larvae, protonymphs, and deutonymphs all declined. The rate on larvae showed the greatest decline (from 43.75% to 18.75%), while that on deutonymphs decreased the least (from 14.29% to 11.76%). At the highest alternative prey density (5 individuals), TU‐strain adult females exhibited relatively low cannibalism rates across all stages (Figure 2A).

When prey density increased from 0 to 5 individuals per arena, the cannibalism rates of the OB‐strain females on eggs, larvae, and protonymphs exhibited a decreasing trend. Specifically, the rate on eggs decreased most markedly (from 50.00% to 23.08%), whereas the rate on larvae changed minimally, remaining nearly constant. Conversely, the cannibalism rate on deutonymphs exhibited an increasing trend (from 6.67% to 26.67%). At the highest alternative prey density (5 individuals), OB‐strain adult females exhibited relatively low cannibalism rates across all stages, except for the larval stage, which remained above 50% (Figure 2B).

### 3.2 Results of GLM Analysis on the Probability of Cannibalism Occurrence

GLM analysis revealed that the main effect of life stage of *N. californicus* was highly significant (*χ* ^2^ = 29.428, df = 3, *p* < 0.001), and the main effect of rearing strain was also significant (*χ* ^2^ = 9.452, df = 1, *p* = 0.002). In contrast, the density of alternative prey had no significant influence (*χ* ^2^ = 0.573, df = 1, *p* = 0.449). Furthermore, a significant interaction was observed between rearing strain and conspecific life stage on the probability of cannibalism (strain × stage: *χ* ^2^ = 8.222, df = 3, *p* = 0.042).

### 3.3 Interaction Between Rearing Strain and Conspecific Life Stage on the Probability of Cannibalism

**FIGURE 3:**
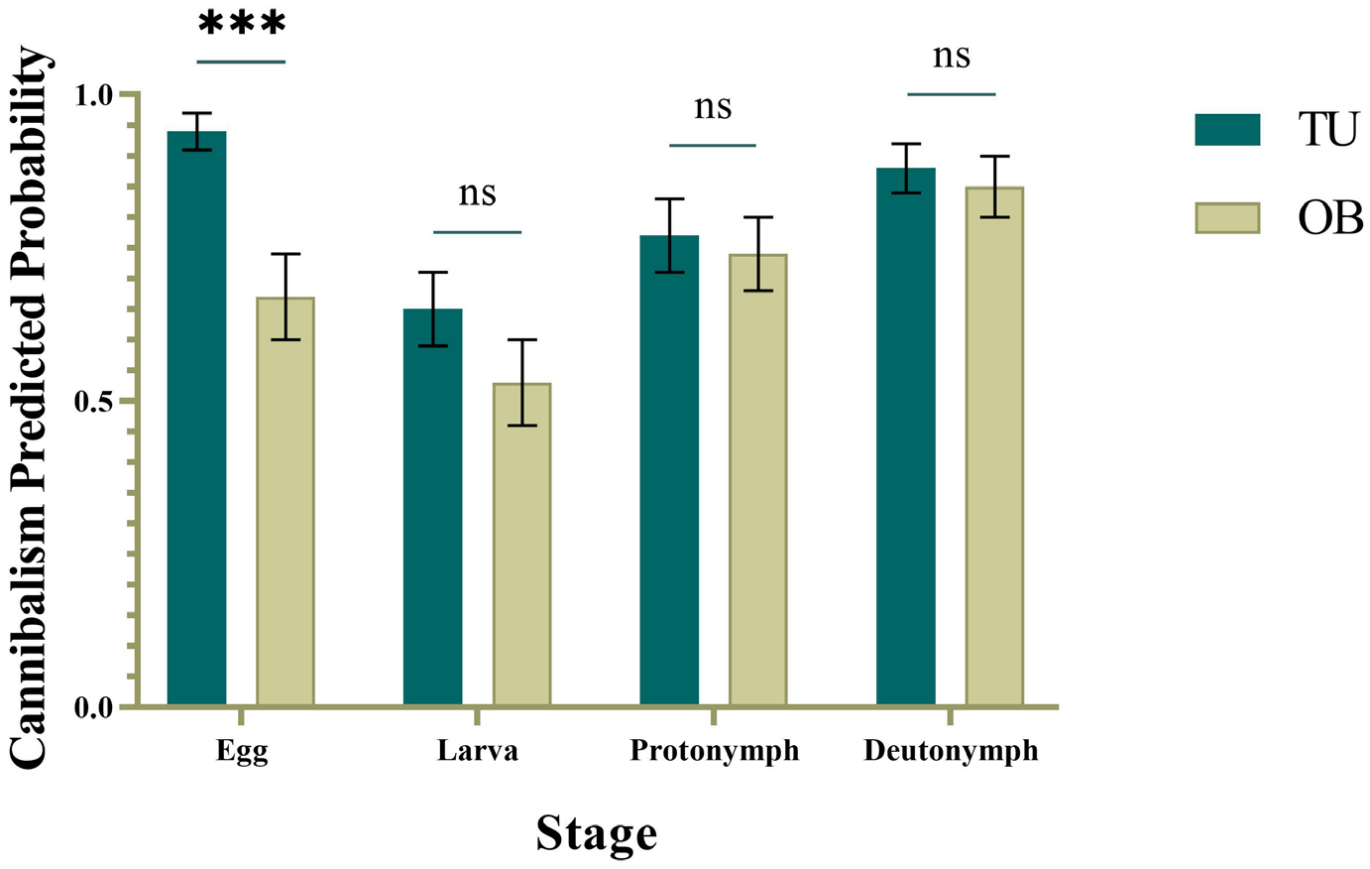
Interaction between strain and life stage on cannibalism probability in *N. californicus* (*** indicates significant differences between strains within a life stage at *p* < 0.05; ns indicates no significant differences between strains within a life stage at *p* > 0.05).

Further analysis of the interaction between rearing strain and conspecific life stage indicated that, at the egg stage, the probability of cannibalism was significantly lower for the OB‐strain than for the TU‐strain (*p* < 0.001). At the other life stages (deutonymph, larva, protonymph), no significant differences were detected between the two strains (*p* > 0.05). However, across these three stages, the probability of cannibalism in the OB‐strain was consistently (though non‐significantly) lower than that in the TU‐strain, with the difference being most evident at the larval stage (Table 2; Figure 3).

**TABLE 2:** Predicted probabilities of cannibalism by adult females of different *N. californicus* strains toward conspecific life stages (*n* = 489).

Stages	TU (Mean ± SE)	OB (Mean ± SE)
Egg	0.94 ± 0.03^a^	0.67 ± 0.07^b^
Larva	0.65 ± 0.06^a^	0.53 ± 0.07^a^
Protonymph	0.77 ± 0.06^a^	0.74 ± 0.06^a^
Deutonymph	0.88 ± 0.04^a^	0.85 ± 0.05^a^

*Note:* Within the same row, different letters indicate significant differences (*p* < 0.05) among strains within the same life stage.

## 4 Discussion

Understanding the effect of prey on cannibalism of *N. californicus* is essential for population maintenance. Therefore, this study focused on the effects of alternative prey ( *O. bakeri* ) density and rearing strain on cannibalism in adult females of *N. californicus* . The results showed that increased alternative prey density generally suppressed cannibalistic behavior, but the magnitude and direction of this effect depended on both rearing strain and conspecific life stage. Rearing strain and life stage were significant factors influencing the probability of cannibalism, with a significant interaction between them. Specifically, the OB‐strain exhibited significantly lower cannibalism than the TU‐strain only toward eggs, while it also showed a lower (non‐significant) trend of cannibalism across other life stages. These findings can directly inform key decisions regarding cost control, strain selection, and rearing management for the mass rearing of *N. californicus* .

The experimental results showed that the density of *O. bakeri* had no significant effect on the cannibalism rates of female adults from both strains (*p* > 0.05). This contrasts with previous studies that found a significant regulatory effect of prey density on cannibalism. For instance, the cannibalism rate in two other phytoseiid mites, *N. neobaraki* and *N. paspalivorus*, decreased significantly in the presence of abundant herbivorous prey (Negloh et al. 2012). Similarly, both the cannibalism and total predation rates of *Agistemus exsertus* (Acari: Stigmaeidae) declined with increasing egg density of *T. urticae* (Rasmy and Saber 2012). Conversely, under conditions of prey scarcity, cannibalism was significantly enhanced in two phytoseiid species ( *N. californicus* and *Typhlodromus bagdasarjani*), and in the thrips *Scolothrips longicornis* (Farazmand et al. 2014). This trend also extends to coleopterans, as prey scarcity increased cannibalism among females of the ladybeetle *Menochilus sexmaculatus* (Coleoptera: Coccinellidae) (Singh et al. 2019). This discrepancy may be due to two factors. First, the alternative prey may be nutritionally inadequate or have limited palatability as a sole food source (Elmoghazy et al. 2024). Although *O. bakeri* can serve as an alternative prey for *N. californicus* (Zhu et al. 2023), its nutritional quality or palatability has been reported to be lower than that of the natural prey *T. urticae* (Mu et al. 2025). Simply increasing the supply of low‐quality alternative prey may not effectively reduce population losses from cannibalism and may instead elevate rearing costs. Second, we examined how predators behaviorally respond to changes in prey density (Murdoch and Oaten 1975). Although statistically non‐significant, a clear trend was observed: with the increase in *O. bakeri* density, the cannibalism rate of the TU‐strain on conspecific larvae showed a decreasing trend (Figure 2A), and that of the OB‐strain on eggs also exhibited a declining trend (Figure 2B). These findings suggest that alternative prey density has a regulatory effect on the cannibalistic behavior of *N. californicus* .

Studies have found that the rearing food source of *M. sexmaculatus* affects its prey preference (Yadav et al. 2023). This aligns with the broader principle that diet shapes predatory behavior. Consistent with this, our study found that adult females of both strains exhibited the highest cannibalism rates toward larvae. This result is consistent with previous research showing that adult females of phytoseiid mites primarily prey on smaller conspecific immature stages and exhibit a stronger preference for larvae during predation (Schausberger 2003). Phytoseiid larvae are small in size, have weak mobility, and are soft‐bodied—traits that make them easy for adult mites to digest. When predatory mites attack larvae, they consume less energy per unit time while achieving a high energy acquisition rate (Schausberger and Croft 1999; Walzer et al. 1999b). Nymphs have larger body sizes, sclerotized cuticle, and greater mobility, which significantly increase handling costs. Therefore, the energy obtained from nymphs is significantly lower than that from conspecific larvae and eggs. Consequently, this drives predators to prefer younger prey (Calabuig et al. 2017). Additionally, studies have shown that *P. persimilis* and *T. bagdasarjani* both exhibit higher predation rates and fecundity when feeding on conspecific larvae (Ghasemloo et al. 2016). The cannibalism rates toward eggs were lower than those toward larvae for females of both strains. This may be because predatory mites are not adapted to piercing oval eggs, and the chorion may possess structural defenses that hinder their feeding behavior (Farazmand et al. 2014).

This study, using the GLM, confirmed a significant interaction between rearing strain and conspecific life stage (strain × stage) on cannibalistic behavior in *N. californicus* , with the OB‐strain showing a significantly lower probability of preying on conspecific eggs compared to the TU‐strain (*p* < 0.001). Previous research has indicated that long‐term rearing of *N. californicus* on *O. bakeri* has a minor impact on its predatory capacity while conferring higher survival rates and favorable oviposition and population growth parameters (Zhu et al. 2022, 2023). However, in dense rearing environments, higher survival rates can intensify intraspecific competition. If poorly managed, this could lead to exacerbated cannibalism, ultimately hindering actual population growth (Revynthi et al. 2018). The significant strain × stage interaction suggests that cannibalism risk is not uniformly distributed. The OB‐strain's low‐risk behavior toward eggs occurs alongside its similar performance to the TU‐strain at other stages. This offers a clear focus for refined rearing management. Consistent with Schausberger's (2003) observation that predatory mites tend to attack vulnerable conspecific stages, the greatest risks in mass rearing are eggs and larvae. Therefore, an optimized rearing strategy for the OB‐strain should take advantage of its low aggressiveness toward eggs by moderately increasing rearing density during population expansion phases. Such stage‐specific, differential management, based on strain behavioral traits, is essential for achieving stable and efficient operation of large‐scale rearing systems.

The unique behavioral pattern of the OB‐strain likely results from behavioral plasticity or genetic adaptation resulting from long‐term exposure to artificial rearing conditions and the specific alternative prey ( *O. bakeri* ). This finding is consistent with Revynthi et al. (2018), who noted that long‐term laboratory rearing alters natural enemy behavior. Unlike the often‐feared behavioral decline, however, the low egg‐predation trait of the OB‐strain identified in this study represents a behavioral characteristic beneficial for mass rearing. This suggests that low aggressiveness toward key life stages could be intentionally used as a breeding criterion. Combined with low‐cost feeds such as *O. bakeri* , this approach allows for the directed development of specialized strains suited to industrial production, thereby reducing reliance on natural prey and supporting an efficient, economical, and sustainable system for large‐scale natural enemy production. Further research is needed to develop standardized rearing protocols and management guidelines for the OB‐strain.

## 5 Conclusions

In summary, this study identified a strain‐specific pattern in the cannibalistic behavior of *N. californicus* that has significant practical implications. Under large‐scale, high‐density rearing conditions, eggs and larvae are the most vulnerable stages. The results show that using the alternative prey *O. bakeri* in large‐scale rearing of *N. californicus* can enhance egg hatchability and larval survival, thereby significantly enhancing population growth efficiency. Adopting the OB‐strain, along with appropriate rearing management, can effectively reduce cannibalism‐related losses, increase production efficiency and population stability, and thus establish a foundation for a sustainable, low‐cost supply of biological control products.

## Author Contributions

**Fan‐Xue Zhang:** conceptualization (equal), data curation (equal), formal analysis (equal), investigation (equal), methodology (equal), software (equal), writing – original draft (equal), writing – review and editing (equal). **Xiang‐Zhi Chen:** data curation (equal), formal analysis (equal), methodology (equal), validation (equal), writing – original draft (equal), writing – review and editing (equal). **Feng Xiao:** conceptualization (equal), formal analysis (equal), methodology (equal), resources (equal), writing – original draft (equal), writing – review and editing (equal). **Jia‐Yun Zhu:** data curation (equal), formal analysis (equal), methodology (equal), writing – original draft (equal), writing – review and editing (equal). **Rong Xiao:** conceptualization (equal), funding acquisition (equal), investigation (equal), project administration (equal), supervision (equal), visualization (equal), writing – original draft (equal), writing – review and editing (equal).

## Funding

The study was financially supported by Guizhou Provincial Science and Technology Projects (Grant Number: Qian Ke He Support [2022] General 135).

## Conflicts of Interest

The authors declare no conflicts of interest.

## Data Availability

All data used in the preparation of this paper are provided in Dryad [https://doi.org/10.5061/dryad.kd51c5bmf].
